# Primary closure of ileostomy site after irrigation with betaine–polyhexanide: a case series

**DOI:** 10.1093/jscr/rjaf846

**Published:** 2025-10-23

**Authors:** Henry Krasner, Abigail W Cheng, Lance Horner, Ovunc Bardakcioglu

**Affiliations:** Kirk Kerkorian School of Medicine at the University of Nevada Las Vegas, 625 Shadow Ln, Las Vegas, NV 89106, United States; Department of Surgery, Kirk Kerkorian School of Medicine at the University of Nevada Las Vegas, 1707 W Charleston Blvd, Las Vegas, NV 89102, United States; Division of Colon and Rectal Surgery, Kirk Kerkorian School of Medicine at the University of Nevada Las Vegas, 1707 W Charleston Blvd, Las Vegas, NV 89102, United States; Division of Colon and Rectal Surgery, Kirk Kerkorian School of Medicine at the University of Nevada Las Vegas, 1707 W Charleston Blvd, Las Vegas, NV 89102, United States

**Keywords:** ileostomy closure, surgical site infections, wound irrigation, primary closure, betaine–polyhexanide

## Abstract

Surgical site infection (SSI) remains a major concern after ileostomy closure, limiting the adoption of primary closure despite the cosmetic and practical advantages of primary closure. A 0.1% betaine–0.1% polyhexanide solution, a surfactant–disinfectant solution with biofilm-disrupting properties, has been shown to significantly reduce SSI rates in various acute and chronic wounds throughout the body. This single-institution case series is the first in the literature to evaluate SSI rates with the use of betaine–polyhexanide irrigation and primary closure of ileostomy sites. All patients demonstrated well-healed incisions without SSI, wound dehiscence, or other wound-related complications at their follow-up visits 10–48 days post-operatively. Compared to the historically high SSI rates with ileostomy site primary closure, this study’s findings suggest that betaine–polyhexanide irrigation may allow for safe primary closure while preserving its advantages of reduced wound care burden and improved cosmesis.

## Introduction

Surgical site infection (SSI) is a well-documented complication following ileostomy closure that increases morbidity, prolongs recovery, increases healthcare costs. Purse-string closure is currently the gold standard for ileostomy closure, given its lower SSI rates compared to primary closure (5% and 29%, respectively) [1–5]. However, purse-string closure results in longer healing time, daily wound care, and more noticeable scarring compared to primary closure [1–5]. Primary closure offers improved cosmesis, minimal to no wound care post-operatively, and faster healing time, but is underutilized due to high SSI rates [1, 3–6]. Various strategies to decrease infection rates with primary closure have yielded mixed results, highlighting the need for alternative approaches [7, 8].

Wound irrigation prior to wound closure is an important component for reducing SSI, as it reduces microbial load [7]. Sterile saline, though widely used for wound irrigation, has been shown to be deficient in adequately disrupting microbial biofilms, with SSI rates of up to 31.6% in ileostomy closure patients with primary closure [9, 10]. Chlorhexidine gluconate is an antiseptic solution that has been shown to reduce SSIs in surgical wounds compared to sterile saline by disrupting microbial biofilms, with one study showing a 13.7% decrease in SSI rate [9, 11–13]. However, its availability is inconsistent and hospital-dependent. A 0.1% betaine–0.1% polyhexanide solution, a surfactant–disinfectant solution, has demonstrated potent ability to disrupt biofilms and efficacy in reducing infection rates in acute and chronic wounds [10]. Studies have shown a decrease in wound infection rates from 40% to as low as 3% with its use, though its use in ileostomy site primary closure has not yet been investigated to the best of our knowledge [10, 14–18]. This case series is the first in literature to examine the novel application of betaine–polyhexanide irrigation in primary closure of ileostomy site in five patients.

## Case series

This is a single-institution retrospective case series conducted in Las Vegas, Nevada. Patients who underwent diverting loop ileostomy reversal with 350 cc of betaine–polyhexanide irrigation followed by primary closure between November 2023 and October 2024 were included (*n* = 5). All patients underwent the appropriate pre-operative evaluation and diagnostics to ensure medical optimization and anatomical appropriateness prior to the reversal. The interval from ileostomy creation to reversal ranged from 2 to 3.5 months (Table 1).

**Table 1:** Summary of patient demographics, clinical characteristics, and postoperative outcomes

	**Age, sex**	**Past medical history**	**Reason for diverting loop ileostomy (DLI)**	**Length of time with DLI (month)**	**Hospital length of stay (days)**	**Time to first post-op follow-up (days)**	**30 days SSI**	**30 days other wound complications**	**30 days readmission or mortality**	**Patient wound-related complaints**
Patient 1	55 M	None	Rectal cancer s/p LAR	3.5	3	10	No	No	No	None
Patient 2	46 M	Asthma, HTN, marijuana use	Diverticulitis s/p sigmoidectomy	3.5	3	28	No	No	No	None
Patient 3	81 M	CHF HFrEF 25%–30%, MI s/p 3-vessel CABG, HTN, CVA, a-fib	Rectal cancer s/p LAR	2.5	8	n/a	No	No	Yes, wound-unrelated cardio-pulmonary arrest	None
Patient 4	50 M	CKD 3, gout	Rectal adenoma s/p LAR	2	3	48	No	No	No	None
Patient 5	93 M	HLD, HTN, diverticulosis	Rectal cancer s/p LAR	3	4	27	No	No	No	None

### Case 1

**Figure 1:**
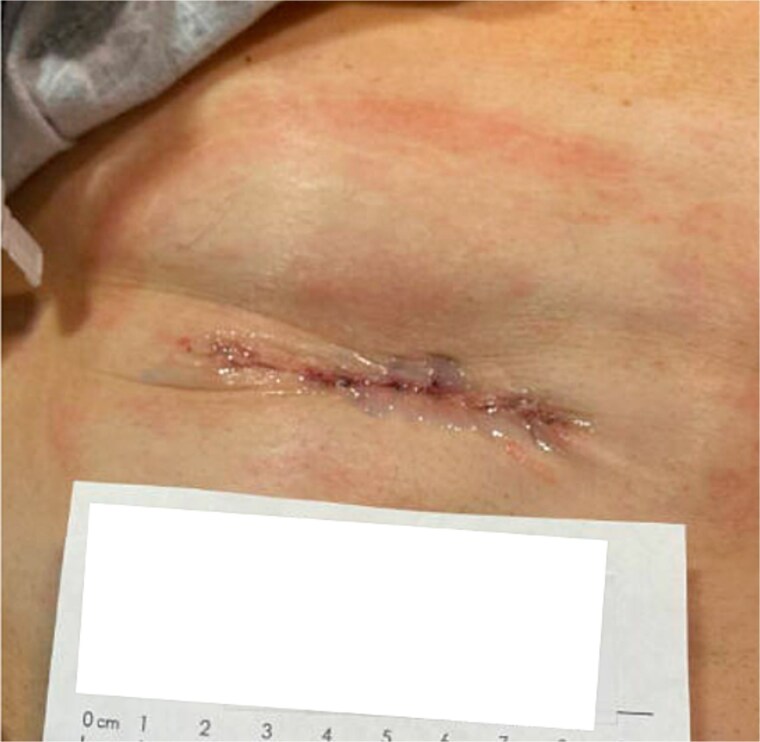
Patient 1 on day of surgery.

A 55-year-old male with history of rectal cancer status post low anterior resection and diverting loop ileostomy creation underwent ileostomy reversal 3.5 months after creation (Fig. 1). His post-operative course was uncomplicated and he was discharged home on post-operative day (POD) 3. At his follow-up appointment on POD 10, the incision was well-healing without signs of infection, wound dehiscence, skin irritation, or other wound-related complications or complaints.

### Case 2

A 48-year-old male with history of diverticulitis status post sigmoidectomy and diverting loop ileostomy underwent reversal 3.5 months after creation. He was discharged home on POD 3 following an uncomplicated post-operative course. At his follow-up appointment on POD 28, the incision was well-healed without any wound complications or complaints.

### Case 3

**Figure 2:**
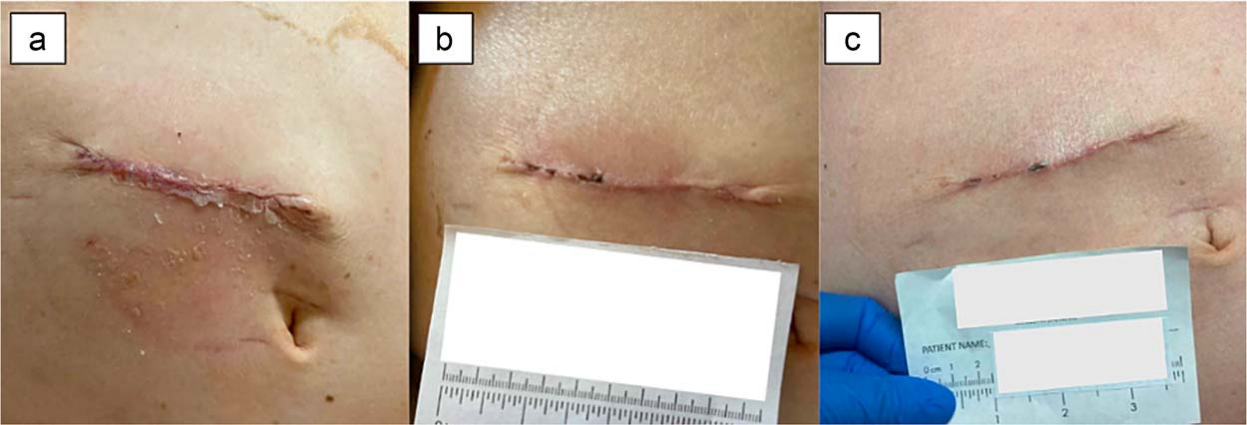
Patient 3 on (a) POD 4, (b) POD 13, (c) POD 26.

An 81-year-old male with history of rectal cancer and significant cardiac comorbidities underwent ileostomy reversal 2.5 months after undergoing low anterior resection and diverting loop ileostomy creation. He had an uncomplicated post-operative course and was discharged home on POD 8. Post-operatively, his incision healed well without any complications (Fig. 2). However, he was readmitted on POD 11 with diarrhea and hypotension, and unfortunately succumbed to cardiopulmonary complications on POD 41.

### Case 4

**Figure 3:**
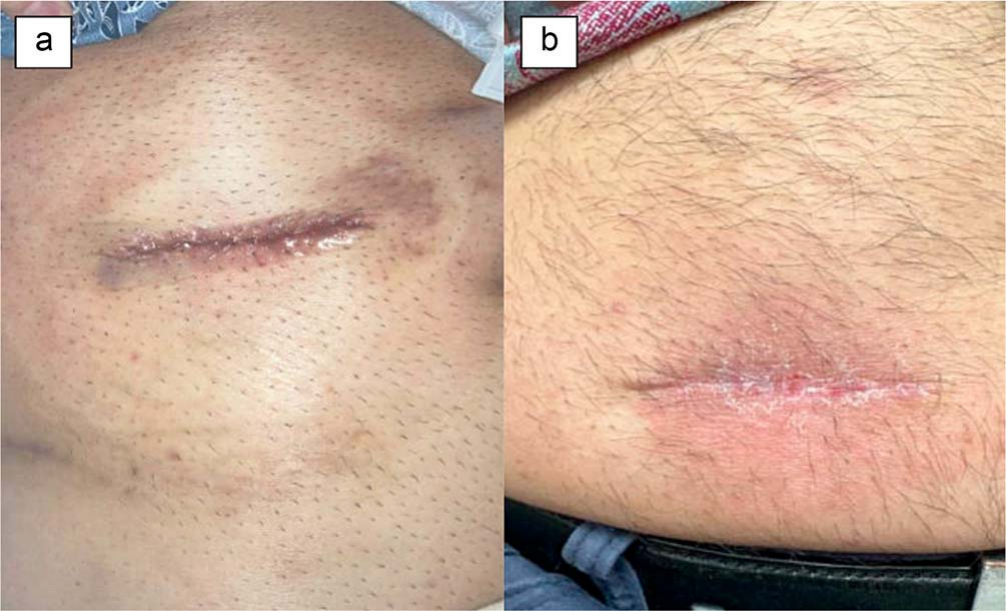
Patient 4 on (a) day of surgery, (b) POD 48.

A 50-year-old male with history of rectal adenoma status post low anterior resection with diverting loop ileostomy creation underwent ileostomy reversal at 2 months after its creation. His post-operative course was uncomplicated and he was discharged home on POD 3. At his follow-up visit on POD 48, the incision was fully healed without any wound complications or patient complaints (Fig. 3).

### Case 5

**Figure 4:**
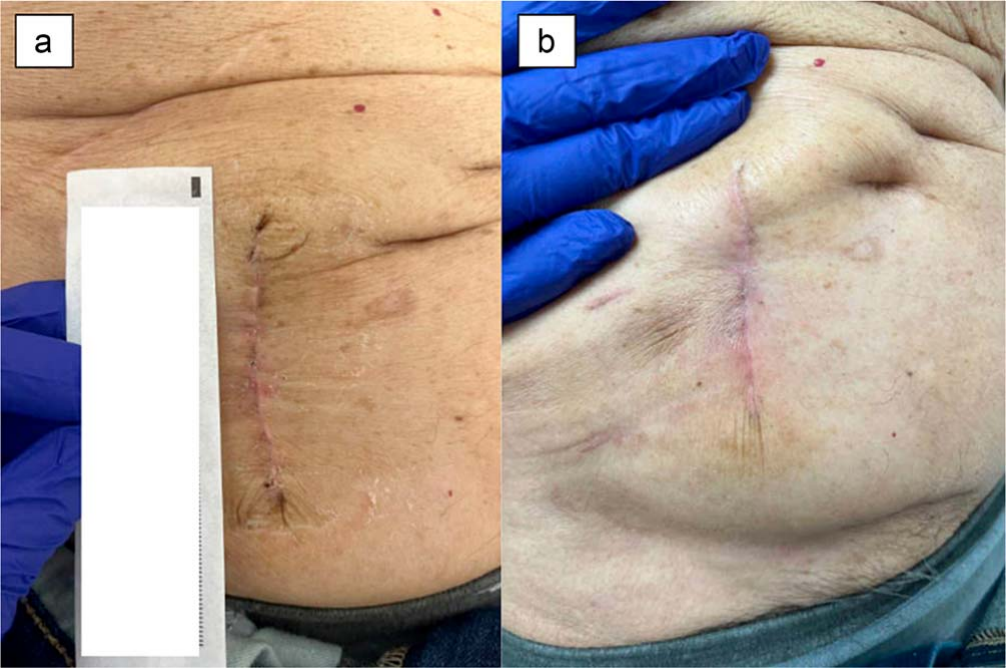
Patient 5 on (a) POD 27, (b) POD 46.

A 93-year-old male with history of rectal cancer status post low anterior resection with diverting loop ileostomy creation who underwent ileostomy reversal 3 months after its creation. Post-operatively, he progressed expectedly and was discharged home on POD 4. At his follow-up visit on POD 27, the incision was well-healed without any signs of infection, dehiscence, or irritation (Fig. 4). The patient was without any complaints.

## Discussion

In this case series, we investigated the association between betaine–polyhexanide wound irrigation in ileostomy site primary closure and SSI rates or other wound-related complications in five patients. All of the patients had well-healing incisions at follow-up without signs of infection, wound dehiscence, or skin irritation, despite some having significant comorbidities, and one patient ultimately expiring due to unrelated cardiopulmonary causes (Table 1).

These outcomes align with prior studies supporting the efficacy of betaine–polyhexanide in reducing infection rates in surgical wounds. As previously mentioned, SSI rates after primary closure for ileostomy sites have been reported to be as high as 29%, compared to 5% observed with purse-string closures [3, 4, 6]. Our small cohort study’s SSI rate of 0% is noteworthy and suggests that betaine–polyhexanide irrigation may be an alternative to chlorhexidine gluconate for ileostomy site irrigation prior to primary closure.

Primary closure offers several benefits to the patient, including elimination of burdensome daily wound care, faster healing times, and improved cosmesis. If SSI rates can be safely reduced using betaine–polyhexanide irrigation, primary closure of ileostomy sites may be more broadly used, which may improve patient satisfaction, recovery speed, and outcomes [1–5].

There are multiple limitations in this study that may have influenced our results. First, the small sample size, single-institution and retrospective design of this study limit its generalizability and introduces bias. Furthermore, the absence of a control group for direct comparison with purse string closure precludes definitive conclusions. Future large randomized clinical trials are warranted to further assess betaine–polyhexanide’s efficacy in ileostomy site primary closure, its cost-effectiveness, and patient-reported outcomes, such as quality of life and scar cosmesis satisfaction.

## Conclusion

This first preliminary case series suggests that betaine–polyhexanide irrigation may reduce the risk of SSI during primary closure of ileostomy sites. All patients demonstrated favorable wound healing without evidence of infection, dehiscence, or skin irritation, supporting the potential use of betaine–polyhexanide as a wound irrigant in ileostomy site primary closure. Future randomized control studies are needed to directly compare betaine–polyhexanide irrigation with primary ileostomy site closure, other solutions, and purse-string closure to validate this study’s findings.
